# Developing a Versatile Arsenal: Novel Antimicrobials as Offensive Tools Against Pathogenic Bacteria

**DOI:** 10.3390/microorganisms13010172

**Published:** 2025-01-15

**Authors:** Junze Ma, Zheng Lu

**Affiliations:** 1 Guangdong Provincial Key Laboratory of Marine Biotechnology, Department of Biology, Institute of Marine Sciences, Shantou University, Shantou 515063, China; 22jzma@stu.edu.cn; 2 Hainan Province Key Laboratory of One Health, School of Life and Health Sciences, Collaborative Innovation Center of One Health, Hainan University, Haikou 570228, China

**Keywords:** CRISPR/Cas, antimicrobial resistance, nano-antimicrobials, antimicrobial polysaccharides

## Abstract

The pervasive and often indiscriminate use of antibiotics has accelerated the emergence of drug-resistant bacterial strains, thus presenting an acute threat to global public health. Despite a growing acknowledgment of the severity of this crisis, the current suite of strategies to mitigate antimicrobial resistance remains markedly inadequate. This paper asserts the paramount need for the swift development of groundbreaking antimicrobial strategies and provides a comprehensive review of an array of innovative techniques currently under scrutiny. Among these, nano-antimicrobials, antimicrobials derived from ribosomal proteins, CRISPR/Cas-based systems, agents that undermine bacterial bioenergetics, and antimicrobial polysaccharides hold particular promise. This analysis gives special attention to CRISPR/Cas-based antimicrobials, scrutinizing their underlying mechanisms, exploring their potential applications, delineating their distinct advantages, and noting their likely limitations. Furthermore, we extend our exploration by proposing theoretical advancements in antimicrobial technology and evaluating feasible methods for the effective delivery of these agents. This includes leveraging these advances for broader biomedical applications, potentially revolutionizing how we confront bacterial pathogens in the future, and laying a foundation for extended research in multimodal therapeutic strategies.

## 1. Introduction

Since Alexander Fleming’s serendipitous discovery of penicillin in 1928, the field of antimicrobials has expanded extensively, unearthing hundreds of different types. These drugs have profoundly impacted public health by combating various bacterial infections and saving innumerable lives. However, their misuse and overuse in human medicine and agriculture have propelled the emergence of antimicrobial-resistant bacteria. Such bacteria evolve mechanisms to counteract these drugs, significantly reducing or nullifying their effectiveness [1]. Importantly, resistance mechanisms existed even before the industrial-scale production of antimicrobials, as evidenced by samples from permafrost cores and other environments contaminated by human activity [2]. These offer insights into pre-antibiotic resistance. The ongoing evolution of resistance is driven by microbial competition for resources, including natural secondary metabolites, similar to today’s synthetic antimicrobials [3]. 

According to the World Health Organization (WHO), the prevalence of drug-resistant bacteria is increasing, particularly in hospital settings and among the general populace [4]. Common culprits include methicillin-resistant *Staphylococcus aureus*, vancomycin-resistant *Enterococcus* sp., drug-resistant *Streptococcus pneumoniae*, and multiple-resistant Gram-negative bacteria, such as *Enterobacteriaceae* [5]. Resistance is increasing across various antibiotics classes: strains of *Streptococci* are showing greater resistance to penicillins; certain *Enterobacteriaceae*, including *E. coli*, are more resistant to cephalosporins; fluoroquinolones like ciprofloxacin and levofloxacin, as well as aminoglycosides like gentamicin, are facing enhanced resistance from bacteria like *Salmonella* sp. and *Pseudomonas aeruginosa* [6].

The WHO estimates that antimicrobial resistance causes approximately 700,000 deaths annually worldwide, a figure projected to rise to 10 million by 2050 [7]. Resistant infections typically require more expensive treatments, result in longer hospital stays, and increase financial burdens on patients. The emergence of drug-resistant bacteria is narrowing the spectrum of effective treatment options, complicating the management and control of common infections, some of which may become untreatable [8].

On the other side, although antimicrobial resistance has been a concern for decades, the strategies to combat it are still limited. Traditional methods of developing new antimicrobials face challenges such as lengthy research cycles, low success rates, high costs, and non-specific targeting that can disrupt microbial balances [9]. Highlighting these concerns, a recent report by the WHO on the state of antimicrobial agents in clinical and preclinical development worldwide reveals that, since July 2017, only 13 new antibiotics have been approved globally. Notably, just two of these represent new chemical classes that may lead to innovative treatments [10]. These hurdles have made it progressively harder to depend solely on discovering new antibiotics to tackle the escalating problem of bacterial resistance. Additionally, the swift emergence of bacterial multiple antimicrobial resistance further complicates endeavors to efficiently manage and control this evolving issue. To address antimicrobial resistance, urgent innovation is required in the development of new antimicrobial strategies [11].

A diverse array of promising antimicrobials, including nano-antimicrobials, ribosomal protein-based agents, CRISPR/Cas-based systems, bioenergetics-targeting drugs, and antimicrobial polysaccharides are currently under development. These innovative approaches necessitate multidisciplinary collaboration and novel thinking to support their progression to large-scale clinical use. This paper provides an in-depth analysis of these novel antimicrobials, particularly emphasizing CRISPR/Cas-based systems. It delves into their mechanisms of action and wide-ranging applications, evaluates their advantages and limitations, and offers recommendations for their refinement. Moreover, the review broadens the scope beyond conventional gene editing roles of CRISPR/Cas to investigate its capabilities in crafting new antimicrobial strategies. This exploration aims to reveal practical implications and foster future interdisciplinary research.

## 2. CRISPR Technology: Reengineering Bacterial Defense for Aggressive Applications

The CRISPR/Cas system is a revolutionary technology that allows us to make precise changes to the DNA of living organisms. Researchers are constantly exploring new ways to harness the power of CRISPR/Cas to offer new insights into diverse challenges and improve our understanding of genetic diseases [12]. Targeting antimicrobial resistance genes using the CRISPR/Cas system is a promising approach to confront the growing issue of antimicrobial resistance. By specifically manipulating genes in bacteria, the CRISPR-based toolkits are possible to disrupt the resistance mechanisms and restore susceptibility to antimicrobials [13].

**Figure 1:**
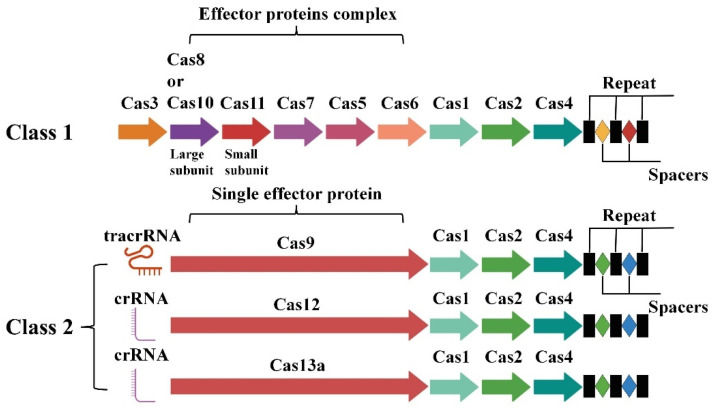
Schematic diagram illustrating the distinctions among CRISPR/Cas system classifications. Cas1: Integrate foreign DNA fragments into the host’s CRISPR locus; Cas2: Collaborates with Cas1 to facilitate the integration of foreign DNA fragments into CRISPR sequences; Cas4: Plays a crucial role in the initial recognition and processing of foreign DNA; Cas5: Contributing to the structural integrity of the complex and engaging in interactions with CRISPR RNA (crRNA); Cas6: Facilitates the production and maturation of crRNA, activation of the CRISPR system; Cas7: Plays a crucial role in binding to crRNA and forming a stable complex; Cas8/10: Directing the Cascade complex to bind to target DNA; Cas11: A multifunctional protein; Large/Small subunit: Large subunits usually house the primary catalytic activity, while small subunits often carry out RNA binding or regulatory functions; Repeat: A short, repetitive DNA sequence; Spacer: A unique DNA sequence situated between the repeat.

The CRISPR/Cas system is categorized into two major classes (Class 1 and Class 2) and six sub-types based on the variation in the Cas proteins. In Class 1 systems, which include types I, III, and IV, effector complexes are composed of multiple proteins that work together to target and cleave the DNA. Class 2 systems, such as type II, V, and VI, on the other hand, rely on a single effector protein for their function. Cas9, which is the most deeply studied and widely used, belongs to type II (Figure 1) [14]. Several CRISPR/Cas systems, including CRISPR/Cas3, Cas9, and Cas13 have been successfully used to specifically eliminate bacteria carrying antimicrobial resistance genes. Cas12, Cas14, and others also have potential as sequence-specific antimicrobials [15]. These systems provide a promising alternative to traditional antimicrobials by providing a highly targeted approach to eliminate specific bacterial gene sequences.

### 2.1. Advantages of Traditional Antibiotics

One of the major advantages of using CRISPR technology in this way is its specificity. Unlike traditional antibiotics, which often indiscriminately affect both harmful and beneficial microbes, CRISPR technology can be tailored to selectively target and eliminate only the harmful bacteria, minimizing collateral damage to the microbiome. Additionally, the adaptability of CRISPR/Cas systems allows for customization to target different environmental bacterial strains, potentially enhancing bactericidal efficacy against a diverse range of pathogens. The precise mechanism of action of CRISPR, which targets nucleic acids within bacteria, differs from traditional antimicrobials that typically disrupt bacterial metabolism or cell wall synthesis, offering a novel strategy to combat antimicrobial resistance and improve treatment outcomes [16]. 

Moreover, CRISPR’s adaptability allows for the rapid development of new therapies in response to emerging resistance patterns. As new genetic mutations responsible for resistance are discovered, new gRNAs can be synthesized to target and neutralize these threats. This flexibility is a significant improvement over the traditional drug development pipeline, which is often lengthy and cannot keep pace with the rapid mutation rates seen in bacteria.

### 2.2. CRISPR/Cas3-Based Antimicrobials

**Figure 2:**
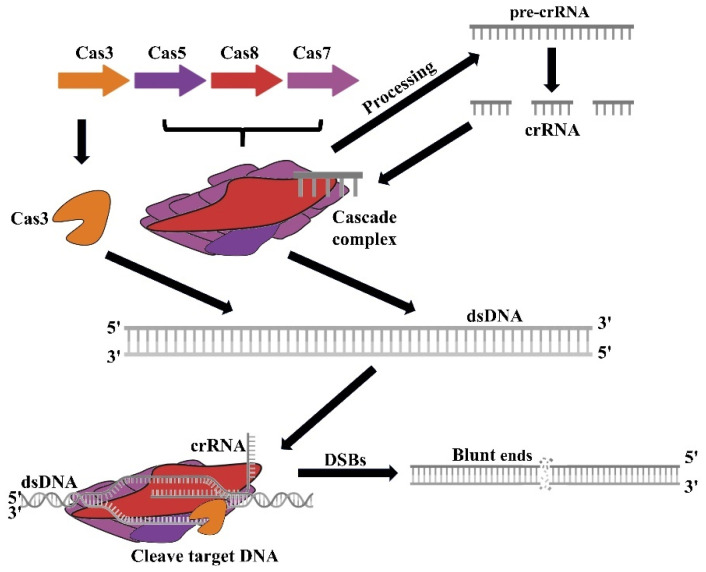
Illustration of the type I-C CRISPR/Cas3 system. Cas proteins process pre-crRNA into mature crRNA. This mature crRNA, along with other Cas proteins, assembles into a complex that directs Cas3 to cleave target sequences. Cas3 induces double-strand breaks (DSBs) in proximity to the target sequence.

The CRISPR/Cas3 system is a Class 1 Type I system, where the Cas3 protein, containing phosphohydrolase and spinase domains, is the labeled protein [17]. This system is prevalent among bacteria and archaea, constituting over 90% of the population. It involves multiple effector proteins that form a cascade protein complex to target DNA; the type I-C CRISPR/Cas3 system’s cascade contains Cas5, Cas8, and Cas7 (Figure 2) [18]. Once bound to DNA, the cascade protein complex recruits Cas3 to cleave the DNA. During the expression stage, Cas proteins process pre-CRISPR RNA (pre-crRNA) into mature crRNA, which, along with other Cas proteins, forms a complex that guides Cas3 to cut foreign target sequences [19]. Unlike other Cas proteins, which merely split the DNA double-strand, Cas3 continuously degrades DNA fragments up to 100 k base pairs in length. Consequently, Cas3’s capacity for extensive DNA degradation provides substantial benefits for large-scale genomic studies [20].

CRISPR/Cas3-based sequence-specific antimicrobials function by breaking double-stranded DNA and are sometimes used in conjunction with other methods to eradicate targeted bacteria. The system specifically targets DNA sequences in a highly precise manner, making it a promising approach for selectively eliminating pathogenic bacteria while sparing beneficial or harmless microorganisms. This targeted approach helps to reduce the development of resistance and minimize off-target effects, thus enhancing the effectiveness of the treatment [21]. For example, by expressing self-targeting CRISPR to redirect endogenous CRISPR/Cas3 activity towards bacterial chromosomes, Kurt Selle et al. were able to effectively target and kill *Clostridioides difficile* both in laboratory settings and in living organisms [22]. Paul Kim and colleagues developed a CRISPR-Cas3-enhanced phage mixture, successfully treating uncomplicated urinary tract infections (UTIs) caused by *E. coli* [23]. Yang et al. investigated the combined effects of CRISPR-Cas3 and restriction-modification (R-M) systems in resisting the transfer of anti-plasmids in Klebsiella pneumoniae. Their findings revealed a significant negative correlation between the presence of these two defense systems and the prevalence of anti-plasmids. Notably, when both systems coexisted within the same host, they effectively blocked the invasion of anti-plasmids and reduced plasmid transfer during conjugation, demonstrating a strong synergistic immunity against plasmid transfer [24].

One notable feature of the CRISPR/Cas3 system is its ability to be adapted. This system can be customized to suit the specific characteristics of different bacteria, which provides us with the flexibility to develop drugs targeting specific resistant strains and efficiently address evolving clinical needs [25]. For example, Zhou et al. utilized a conjugative endogenous CRISPR-Cas3 system to demonstrate high efficiency in curing antimicrobial-resistant plasmids in both in vitro and in vivo infection models. This highlights its potential for using native CRISPR-mediated plasmid curing to re-sensitize drug-resistant *K. pneumoniae* to multiple antibiotics [26]; By incorporating the IE-type CRISPR-Cas3 system from *Escherichia coli BW25113* into *Escherichia coli Nissle 1917 (EcN)*, Fang et al. successfully equipped *EcN* to effectively target and cleave multiple antibiotic resistance genes (ARGs). This approach significantly minimizes the risk of resistance gene transmission when *EcN* is used as a probiotic. Moreover, it was observed that the type I CRISPR-Cas system imposes a lower growth burden compared to the type II system, offering better prospects for clinical applications [27]. Compared to other Cas proteins, the long DNA degradation capability of Cas3 is particularly advantageous for large-scale genomic studies. Furthermore, the CRISPR-Cas3 system originated as a bacterial immune mechanism, allowing it to adapt and respond to various types of viruses, which enhances its versatility.

There are several challenges and limitations associated with this type of antimicrobial agent. The efficacy of targeting bacteria with plasmids that carry antimicrobial resistance genes can be compromised if the cleavage of plasmid DNA does not lead to bacterial death [28]. Additionally, the complexity of CRISPR/Cas3 technology, relative to other systems such as CRISPR/Cas9, necessitates specialized knowledge and equipment, which can pose significant challenges. The CRISPR/Cas3 system is primarily found in a limited number of specific bacteria and archaea, exhibiting greater diversity and complexity in the CRISPR loci and Cas gene families within their genomes. In contrast, systems like Cas9 and Cas12 are more prevalent across many bacterial species and are relatively straightforward in both structure and function [29]. The off-target effects of the CRISPR/Cas3 system and the efficient delivery of it in complex microbial environments present significant challenges. When target sequences closely resemble non-target sequences, the Cas3 protein may inadvertently recognize and cleave these non-target regions [30]. This can lead to unintended genomic changes, resulting in phenotypic alterations or cell dysfunction. Additionally, prolonged sequence interactions can activate Cas3 inappropriately, raising the risk of off-target effects [31]. Such effects may trigger a cell’s DNA damage response, potentially leading to apoptosis or other negative consequences. Using CRISPR/Cas3 technology in intricate settings, like the gut microbiome, poses challenges due to the high genomic diversity and similarity among microbes, which can result in insufficient selectivity [32]. Effective delivery of CRISPR/Cas3 systems to target microbes is also problematic in these environments, as biofilms produced by certain microorganisms can impede the entry of foreign DNA [33]. Therefore, developing reliable delivery mechanisms (such as nanoparticles or viral vectors) is crucial. Furthermore, the large-scale application of CRISPR/Cas3 technology could have unforeseen consequences for ecosystems, potentially altering microbial community structures and their functions [34]. As such, thorough ecological risk assessments are essential to ensure safety [35]. There are also safety concerns associated with the use of CRISPR/Cas3, as the full implications of its application in antimicrobials are still under investigation.

Future research in this area could focus on improving accuracy, reducing costs, and enhancing safety to minimize effects on non-targeted bacteria and mitigate potential risks. It is important to continue exploring and addressing these challenges to further advance the effectiveness and safety of CRISPR/Cas3-based antimicrobial strategies.

### 2.3. Programmable Removal of Target Genes Using CRISPR/Cas9

**Figure 3:**
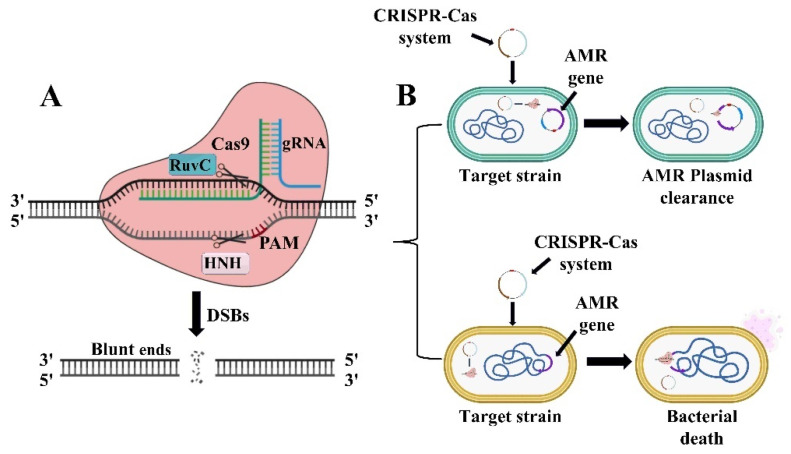
Schematic illustration of CRISPR/Cas9 system action mechanisms. (**A**) The programmed gRNA binds to the Cas9 protein, activating it and transforming it from its inactive to active form. Once activated, Cas9 searches for its target sequence by locating a matching PAM sequence (5′-NGG-3′). Upon identification, Cas9 induces double-strand breaks (DSBs) at three base pairs upstream of the PAM, utilizing its HNH and RuvC domains; (**B**) The outcome of the system is influenced by the location of the target Antimicrobial-resistance (AMR) gene. Cleavage of chromosomal sequences by CRISPR/Cas can result in the death of the targeted bacteria, whereas targeting a sequence on a plasmid can lead to the loss of the plasmid from the host cell.

The CRISPR/Cas9 system, classified within the class 2 type II system, stands as the most thoroughly investigated and commonly utilized CRISPR/Cas module, driven by a solitary effector protein [36]. This system operates through crRNA, which specifically binds to a long RNA molecule known as tracrRNA. The tracrRNA contains a domain that attaches to the Cas protein and a sequence complementary to the repeating region of crRNA, facilitating pairing. The tracrRNA/crRNA complexes are essential in directing the nuclease activity of Cas9 proteins to crRNA-matched sites on double-stranded DNA, culminating in cleavage [37]. Engineers can synthetically create a single-guide RNA (sgRNA) from these two RNAs to simplify the system and enhance Cas9’s targeting accuracy. The sgRNA directs Cas9 to specific DNA cleavage sites, maximizing the system’s functionality [38]. Crucially, the Cas9 protein itself features two key domains: the RuvC domain at the amino terminal and the HNH domain situated centrally. The HNH domain is responsible for cleaving the DNA strand that pairs with crRNA, whereas the RuvC domain cleaves the opposing, non-complementary strand, both crucial for the effective maturation of crRNA and the cleavage of double-stranded DNA (Figure 3) [39].

The mechanism of CRISPR/Cas9-based programmable removal of target genes involves utilizing RNA-guided nuclease Cas9 to induce cell death in recipient bacteria through chromosome lysis or by using nuclease 9 to excise target genes from plasmids [40]. He et al. have made an advancement in CRISPR/Cas9 technology by developing a system that can specifically target and eliminate plasmids carrying antimicrobial resistance genes. In their study, they developed a CRISPR/Cas9 system enclosed within an ISApl1 element and located on a suicide plasmid. This system is capable of specifically targeting and eliminating plasmids harboring the target gene, as well as killing strains that carry the target gene on their chromosome [41]. Rodrigues et al. introduced a CRISPR/Cas system adapted to a signaling response-coupled plasmid, which effectively transfers to Enterococcus faecalis to selectively eliminate antimicrobial resistance genes [42]. Citorik et al. demonstrated that a phage plasmid containing the M13 single-stranded phage packaging signal could effectively deliver the RNA-guided nuclease Cas9 to *E. coli*. This method led to the targeted elimination of pathogenic *E. coli* strains expressing specific target genes, including those associated with antimicrobial resistance. However, some “escape” colonies managed to evade treatment; these colonies were not the result of genetically resistant mutants. Instead, they either did not receive the Cas9 plasmid or were exposed to a defective plasmid [43]. Ates et al. demonstrated that by targeting resistance genes such as *mecA*, *aacA*, *grlA*, and *grlB*, CRISPR-Cas9 can reduce the expression of these genes and reverse the resistance of methicillin-resistant *Staphylococcus aureus* (MRSA) to multiple antibiotics. This study highlights the potential of this technology to restore the effectiveness of antibiotics [44]. Zhang et al. developed an engineered conjugated system utilizing the CRISPR/Cas9 technology to re-sensitize *E. coli* that were resistant to tetracycline and polymyxins. Their approach significantly decreased the population of tetracycline- and polymyxin-resistant bacteria in the body to just 1%. This work opens up new possibilities for creating CRISPR-based tools aimed at selectively eliminating bacterial pathogens and precisely modifying microbiome composition [45].

They can precisely target bacterial genes, decreasing bacterial resistance to antimicrobials and prolonging their efficacy compared to traditional antimicrobials.

Similar to the CRISPR/Cas3 system, CRISPR/Cas9-derived antimicrobials can effectively kill target bacteria only when aiming at genes present within the recipient cell’s chromosome [28]. The limitations of CRISPR/Cas9-based sequence-specific antimicrobials primarily stem from the fact that their application in the realm of antimicrobials is still in the early stages, requiring further research and validation. There is a concern that the CRISPR/Cas9 system may induce unintended changes to the host cell’s genome, necessitating thorough safety assessments [46]. CRISPR/Cas9 technology has emerged as a powerful gene editing tool, but it faces several regulatory and safety challenges in its application. Legal and ethical standards for gene editing differ significantly across countries, complicating international collaboration and consistency in research [47]. Additionally, the approval process for new technologies is often lengthy and involves rigorous clinical trials and ethical reviews, which can delay the clinical application of CRISPR [48]. For human therapies in particular, regulators must ensure the safety and efficacy of gene editing techniques, adding complexity to the approval process [49]. Research has shown that CRISPR/Cas9 can tolerate some mismatches between the guide RNA and target DNA, leading to off-target editing, which poses a significant challenge in clinical applications of the system [50]. As a result, optimizing the Cas9 protein to enhance target specificity and improve clinical safety is a major focus. Additionally, investigating the development of advanced delivery vectors, such as liposomes, may enhance the efficiency and accuracy of the CRISPR/Cas9 system in bacterial applications while minimizing off-target effects [51]. Moreover, introducing foreign CRISPR components into the body may trigger an immune response, further complicating safety and efficacy. When applied to humans, it is crucial to consider the host’s immune system to avoid adverse reactions [52]. While CRISPR/Cas9 holds immense promise, addressing these regulatory hurdles and safety concerns is vital for its responsible application. The scientific community, regulators, and the public must collaborate to ensure its safe and effective use across various fields, from medicine to agriculture.

Compared to other systems, the composition of the Cas9 system is relatively straightforward, and its specific mechanism is well understood. The Cas9 protein alone is capable of performing multiple functions. Future research on CRISPR/Cas9-based sequence-specific antimicrobials should focus on broadening the target spectrum and enhancing the overall utility of the CRISPR/Cas9 system to effectively combat a wider range of bacterial strains. Some Cas9 variants have shown improved specificity and reduced off-target activity during genome editing. Future efforts could prioritize screening and optimizing these variants to identify the most effective Cas proteins for antimicrobial therapy.

### 2.4. CRISPR/Cas12: Minimal Components, Flexible Antimicrobial Approach

**Figure 4:**
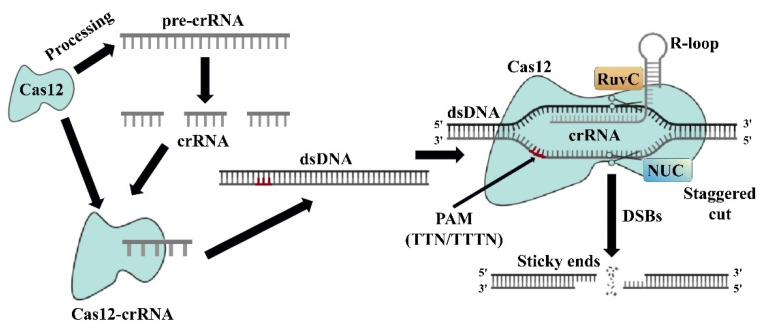
Schematic representation of the CRISPR/Cas12 system. The Cas12 protein requires only crRNA to induce double-strand breaks (DSBs). Cas12 cleaves the target region adjacent to a PAM sequence (TTN/TTTN) using its RuvC and nuclease lobe (NUC) domains. When Cas12 encounters its target, it begins the formation of an R-loop, establishing base–pair hybridization between the crRNA and the target DNA strand. During this process, Cas12 aligns with approximately 17 bp of the target sequence, leading to R-loop formation. Once the R-loop is established, the Cas12 protein utilizes its active RuvC domain to create a staggered cut in the non-target strand, guided by the PAM sequence.

The CRISPR/Cas12 system is a Class 2 type V system that utilizes a single effector protein, Cas12, to process crRNA and target specific DNA sequences [53]. Unlike other CRISPR systems that require both crRNA and tracrRNA, Cas12 functions with crRNA alone as a guide for DNA editing. This system involves the Cas12 protein forming an R-loop structure with the target DNA strand, leading to the identification and cleavage of the target DNA sequence. Cas12 carries out DNA cleavage with its RuvC and nuclease lobe (NUC) domain (The NUC domain is responsible for the DNA cleavage activity of the Cas12 protein, working in conjunction with the RuvC domain to ensure efficient cutting of the target DNA), similar to the Cas9 system [54]. The recognition of a target sequence near a PAM sequence by Cas12 triggers the formation of the R-loop and subsequent DNA cleavage at the non-target strand, facilitated by the PAM sequence (Figure 4). However, the function of the RuvC domain of the Cas12 protein in cutting the target DNA strand has not been clearly studied [55].

The compact structure of Cas12, in contrast to Cas9, enhances its efficiency for intracellular delivery, potentially minimizing the immune response and improving its safety for various applications. Furthermore, Cas12 has more flexible target sequence requirements than Cas9, enabling it to recognize and cleave a broader range of DNA sequences. This versatility significantly expands the potential applications of the CRISPR/Cas12 system [56].

At present, there are no reports of CRISPR/Cas12-based sequence-specific antimicrobials [57]. The author believes that if CRISPR/Cas12-based sequence-specific antimicrobials are successfully developed, their mechanism of action should be derived from the characteristics of the broken DNA. This can be achieved by designing specific sgRNA to locate the nucleic acid-protein complex to a specific site of DNA to play its cutting role. If the rate of broken DNA is faster than the rate of cell repair DNA, leaving the DNA of the targeted recipient cell in a broken state will lead to the death of the recipient bacteria. Similarly, due to the action principle of CRISPR/Cas12-based sequence-specific antimicrobials, they can exert effective antimicrobial effects only when the targeted gene is located on the chromosome of the recipient cell [28]. One limitation of the system is its low editing efficiency observed in some studies, which poses a challenge to its broader application [58]. In the future, related applications could be combined with other treatments to further improve efficacy. The efficient delivery of incoming targeted receptor bacteria by the CRISPR/Cas12 system is the primary issue that needs to be addressed to control and prevent antimicrobial resistance of corresponding bacteria by using this system [59]. If efficient and fast delivery methods can be developed, the practical value of CRISPR/Cas12-based sequence-specific antimicrobials will be greatly enhanced. However, further research and experimental validation of the technology is needed to ensure its safety and efficacy.

In conclusion, although relevant studies on CRISPR/Cas12-based sequence-specific antimicrobials have not yet been reported, such studies will eventually be realized as science and technology continue to advance. This will provide humans with an entirely new antimicrobial strategy that promises to change the way we fight bacterial infections [60].

### 2.5. CRISPR/Cas13: RNA-Targeted Bacteria Killers

**Figure 5:**
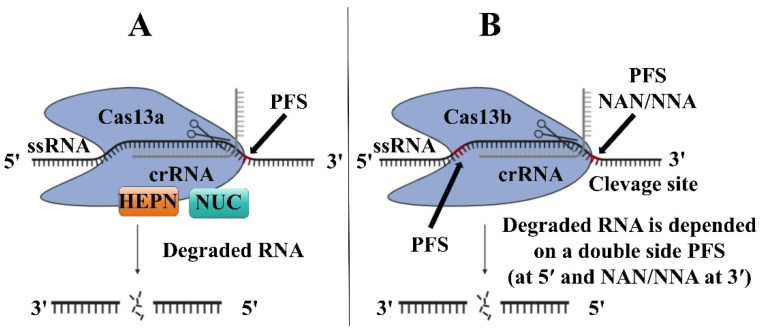
Distinct RNA degradation patterns of two CRISPR/Cas13 systems. (**A**) Cas13a protein is activated by a single crRNA and consists of several components, including the crRNA itself, nuclease (NUC) lobes, and two nucleo-tide-binding (HEPN) RNase domains that enable it to target RNA. Cas13a cleaves ssRNA upon recognizing a target sequence that is 22 to 28 nt long and complementary to the crRNA spacer. This target sequence is flanked by a protospacer-flanking site (PFS) at the 3′ end, allowing crRNA to bind and cleave the target region of ssRNA without the need for tracrRNA; (**B**) Cas13b protein is associated with mature crRNA, forming a complex that searches for target single-stranded RNA (ssRNA). This CRISPR/Cas13b complex induces precise conformational changes in the ssRNA target with the assistance of the Protospacer Fanking Site (PFS), which flanks RNA targeting at the 5′ end and PAM sequence (NAN/NNA) at the 3′ end, resulting in non-specifc RNA cleavage.

The Cas13 protein family contains two subtypes: (1) Cas13a, which comes from the *Leptotrichia shahii* bacterium (LshCas13a), officially named C2c2, and belongs to the type VI CRISPR/Cas system; (2) Cas13b, which comes from *Prevotella* sp. (PspCas13b) belongs to the type III CRISPR/Cas system. The system only targets and cuts single-stranded RNA(ssRNA), not single-stranded DNA(ssDNA) or double-stranded DNA(dsDNA) [61]. The Cas13a protein is activated by a single crRNA, following a similar activation mechanism to the processing of the Cas12 protein. Cas13a proteins include crRNA, NUC lobes, and two higher eukaryotes and prokaryotes nucleotide-binding (HEPN) RNase domains for targeting RNA (Figure 5A) [62]. The Cas13b protein is more precise than the Cas13a protein because the protospacerfanking site (PFS) targets RNA of A, U, or G at the 5′ end, and targets PAM at the 3′ end, and the transcript source is determined by the sequence at the 3′ end of the RNA target site (called PFS) [63]. The Cas13b protein is associated with mature crRNA. The CRISPR/Cas13b complex searches for the target ssRNA and induces conformational changes in the ssRNA target, leading to non-specific RNA cleavage (Figure 5B) [64].

CRISPR/Cas13-based sequence-specific antimicrobials exhibit a unique mechanism of action that distinguishes them from other CRISPR-derived bacteria-killing systems. They are specifically engineered to target RNA, making them particularly effective for certain applications. In contrast, other CRISPR systems primarily focus on DNA, which allows the CRISPR/Cas13 system to avoid making permanent changes to the DNA in some cases [65]. CRISPR-Cas13a has promiscuous ssRNA cleavage activities and restricts host bacteria growth due to the degradation of the bacterial RNAs [66]. This characteristic enables them to effectively fight against bacterial infections, regardless of whether the targeted gene is located on the bacterial chromosome or a plasmid. Moreover, these antimicrobials have shown a high level of efficacy and are capable of overcoming drug resistance. They are adept at targeting bacteria, rapidly killing them, and successfully preventing the development of bacterial resistance, making them a promising alternative to traditional antimicrobials [67].

The research on this group of antimicrobials has made significant strides. Kotaro Kiga et al. developed CapsidCas13a(s), a series of antimicrobial nucleocapsids based on CRISPR/Cas13a [68]. These CapsidCas13a constructs are created by encapsulating programmed CRISPR/Cas13a into a phage nucleocapsid. They can target and eliminate specific antimicrobial genes associated with carbapenem-resistant *E. coli* and methicillin-resistant *Staphylococcus aureus* with precision. Notably, unlike Cas9-based antimicrobials, CapsidCas13a(s) demonstrate potent bacteria-killing activity even when the target gene is located on a plasmid [68]. In another breakthrough, Song et al. devised a strategy leveraging bacterial conjugation to deliver CRISPR/Cas13a for the targeted elimination of *Salmonella typhimurium*. This system relies on crRNA targeting endogenous transcripts of pathogens for precise killing, showcasing efficacy in both laboratory and animal experiments. The method can be readily delivered through bacterial conjugation and holds promise for targeting a range of pathogens. With further refinement and enhancement, this system could be harnessed for microbiome modification and biotherapy applications [69]. Ho-Min Park and colleagues have enhanced the effectiveness of CRISPR/Cas13-based antimicrobials by optimizing the RNA–protein interactions of crRNA and Cas13 proteins. Their research uncovered a significant proportion of Cas13 systems that lack colocalized CRISPR arrays, potentially resulting in suboptimal RNA–protein interactions in current tools due to the absence of a direct association between crRNA and Cas proteins. Through the optimization process, they identified several candidate crRNAs with improved docking capabilities to the current Cas13 protein. This study simplifies the optimization of RNA–protein interactions, which is a crucial initial stage in the development of efficient CRISPR/Cas13-based antimicrobials [70]. Li et al. developed CRISPR-Cas13a-loaded antibacterial capsids (AB-capsids) to combat multidrug-resistant *Staphylococcus aureus*. These AB-capsids can selectively target and eliminate strains of *Staphylococcus aureus* that carry specific target genes while leaving non-target strains unharmed [71].

CRISPR/Cas13-based sequence-specific antimicrobials hold great potential, but they also face significant limitations and clinical challenges. While the CRISPR/Cas13 system is known for its high specificity, there are instances where it can inadvertently recognize and cleave non-target RNAs, leading to off-target effects that may adversely affect host cells [72]. Additionally, pathogens can develop resistance to CRISPR/Cas13 treatments by mutating the target RNA sequence [12]. Furthermore, since Cas13 targets RNA rather than DNA, its effects may be less durable due to the inherent instability of RNA, which could diminish its efficacy and persistence in the body [70]. The process of obtaining regulatory approval for clinical applications is also rigorous and can hinder the promotion and implementation of research findings. Conducting large-scale clinical trials to assess the efficacy and safety of these antimicrobials presents numerous challenges as well [73]. In summary, while CRISPR/Cas13-based antimicrobials offer promising prospects in research, translating them into clinical practice requires overcoming various obstacles. Future studies should focus on improving specificity, addressing resistance, and optimizing delivery systems to enhance their clinical viability.

### 2.6. Potential Development of Antimicrobials Using CRISPR/Cas14

**Figure 6:**
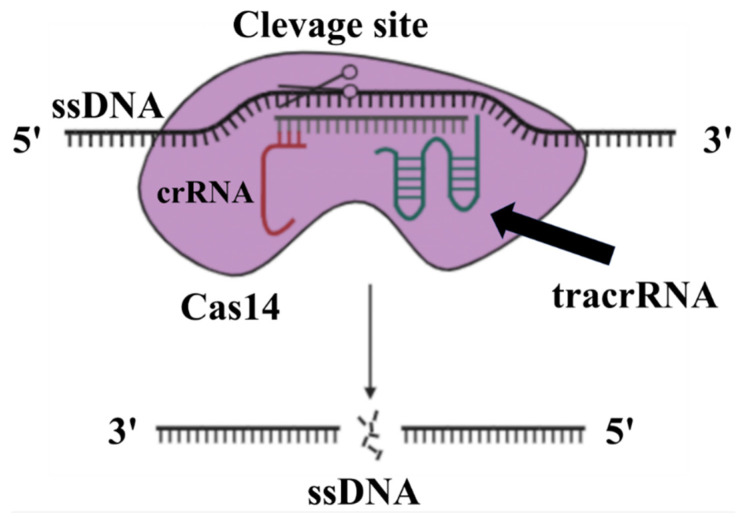
Representation of targeting and cleavage principles in CRISPR/Cas14 System. The Cas14 protein consists of both tracrRNA and crRNA, enabling it to target ssDNA. It recognizes single-stranded DNA with the assistance of tracrRNA and crRNA, mediates interaction with the target ssDNA through the seed sequence, and efficiently cleaves ssDNA while showing no activity against dsDNA or ssRNA. Unlike Cas9, Cas12, and Cas13 proteins, Cas14’s cleavage efficiency is more specific and does not require the presence of a PAM region.

The effector protein of the CRISPR/Cas14 system is the Cas14 protein, which is notably smaller than other identified Cas proteins, with a length of only 400–700 amino acids. Similar to Cas9, the Cas14 protein relies on tracrRNA and crRNA to target DNA. It recognizes ssDNA, facilitates the interaction of the seed sequence with the target ssDNA, and cleaves ssDNA rather than dsDNA or ssRNA [74]. Cas14 proteins utilize a distinct targeting mechanism compared to other Cas proteins, as they can directly recognize and bind to ssDNA without the need for extensive PAM [75]. Their smaller size may contribute to greater flexibility, enabling them to dynamically adapt to the shape and structure of the target DNA. This inherent flexibility allows Cas14 to effectively bind to ssDNA under more permissive conditions, broadening their potential applications, and exhibiting a more specific cutting efficiency compared to Cas9, Cas12, and Cas13 proteins, meeting the criteria for high-fidelity gene editing (Figure 6) [76]. Its small size allows for easier delivery to the target tissue [77]. In the absence of PAM regions, the Cas14 protein is capable of editing dsDNA, offering several advantages over earlier Cas proteins. This feature facilitates manipulation and integration in gene editing and biotechnology applications.

The CRISPR/Cas14 system also has its limitations. One of the key challenges is that the mechanisms of the Cas14 system may be more restricted, particularly in complex genomes where identifying suitable and effective targets can be difficult [78]. Additionally, delivering the CRISPR/Cas14 system into target cells poses a significant technical hurdle, especially in vivo, where optimizing the selection of delivery vectors and improving transduction efficiency is crucial [79]. Like other gene-editing technologies, CRISPR/Cas14 also faces ethical and legal challenges, necessitating careful consideration of existing laws, regulations, and public acceptance [79].

Research on the CRISPR/Cas14 system is still in its early stages. While no studies on CRISPR/Cas14-based sequence-specific antimicrobials have been reported, it is probable that such studies will emerge in the near future [80]. The author suggests that if CRISPR/Cas14-based sequence-specific antimicrobials are successfully developed in the future, their mechanism of action will differ substantially from other CRISPR/Cas-based antimicrobials. Unlike their counterparts, CRISPR/Cas14 can efficiently bind ssDNA under loose conditions without the need for a PAM. This flexibility makes them much less restrictive in their capabilities [81]. Due to the flexible action principle of CRISPR/Cas14-based sequence-specific antimicrobials, they can be applied to a variety of complex situations, no matter where the target is located, it can still effectively play a role in fighting bacteria, particularly for those target cells unable to determine the PAM region [28]. If this technology can be successfully applied to antimicrobial research and development, it will provide a new approach to combat bacterial infections and antimicrobial resistance.

Each of the CRISPR/Cas systems has distinct characteristics that influence their effectiveness and feasibility in combating microbial infections. In brief, the advantages of the Cas9 system lie in its precision and simplicity. Cas9 is highly regarded for its accuracy in targeting specific DNA sequences, which is essential for addressing bacterial genes linked to virulence and antibiotic resistance [82]. Furthermore, its straightforward design and the extensive body of research surrounding it facilitate its implementation and modification [83]. For Cas12, the most appealing features are its broader targeting range and collateral cleavage ability. Cas12 recognizes a wider array of PAM sequences compared to Cas9, enabling it to target a more diverse set of genomic sites across various bacterial species. Additionally, it can non-specifically cleave single-stranded DNA, a capability that may be leveraged to enhance antimicrobial effects [84]. The benefits of the Cas13 system include RNA targeting and the potential for live diagnostics. This protein is distinctive in its ability to target RNA, offering a strategy to combat RNA-based viruses and bacteria where controlling transcription is critical [85]. Additionally, its activity can be exploited for the live detection of pathogen RNA, merging therapeutic and diagnostic functionalities [86].

On the other side, these systems each have their own highlighted disadvantages. Cas9: (1) off-target effects: While precise, Cas9 can still cause unintended effects in the genome, which may pose risks of horizontal gene transfer or mutation in non-targeted bacteria [87]. (2) DNA targeting: Cas9’s restriction to DNA can be a limitation when targeting RNA viruses or RNA-based mechanisms within microbes [88]. Cas12, limited control over collateral activity: the non-specific cleavage, while potentially useful, can also lead to unintended genomic alterations that could complicate therapeutic applications. Cas13, lack of established frameworks [89].

Different CRISPR/Cas systems offer exciting possibilities for developing new antimicrobials. The high-throughput screening capabilities of CRISPR technology can significantly accelerate the discovery of novel antimicrobial agents [90]. Researchers can quickly identify which targets most affect bacterial survival and select various Cas systems according to the specific target and its mechanism, optimizing antibacterial strategies against particular pathogens [91]. Given the growing issue of antibiotic resistance, leveraging these advanced gene-editing tools to explore new antimicrobial options is especially crucial.

### 2.7. Prospects for Other CRISPR/Cas Systems as Sequence-Specific Antimicrobials

In the past few years, the diversity of CRISPR/Cas systems offers a wide range of options for developing sequence-specific antimicrobials tailored to target specific pathogens [32]. Among the six main types of CRISPR/Cas systems (types I-VI), type II, type V, and type VI CRISPR/Cas systems have garnered significant attention and are widely employed in scientific research. These systems are associated with the representative proteins Cas9 (type II), Cas12 (type V), and Cas13a (type VI), which have been extensively studied for their gene editing and nucleic acid-targeting capabilities [92]. Although type I and type III CRISPR/Cas systems exist, research on these systems is not as extensive as that on types II, V, and VI [93]. Type I and type III systems have distinct features and functions that warrant further exploration to better understand their potential applications in genetic manipulation and antimicrobial strategies. Type IV CRISPR/Cas systems, on the other hand, are less well-characterized, and our current understanding of their mechanisms and potential applications is limited. Continued research efforts are needed to elucidate the properties and functions of type IV CRISPR/Cas systems and assess their feasibility for various biomedical and biotechnological applications.

Redondo et al. delved into the least understood and most enigmatic type IV CRISPR/Cas system. A comprehensive analysis of their CRISPR spacer content revealed these systems have a pronounced inclination toward targeting other plasmids. Their data suggests that type IV CRISPR/Cas systems function differently from other host-associated CRISPR/Cas immune systems and play a crucial role in mediating conflicts between plasmids [94]. Plasmids are a distinct form of genetic material reliant on host bacteria for survival. In other words, they are an alternative form of bacterial parasite [95]. Some research indicates that certain plasmids can utilize the Type IV CRISPR/Cas system to counter competition from other plasmids for the same host bacteria. This study presents a paradigm-shifting perspective, demonstrating that humans are not the first to leverage CRISPR/Cas systems as tools [96]. It is important to note that plasmids facilitate the transmission of drug-resistance genes, contributing to the rise of antimicrobial-resistant bacteria and overwhelming challenges in clinical treatment [97]. The findings offer potential solutions in the battle against antimicrobial-resistant bacteria. In the context of escalating antimicrobial resistance in global health, the use of the Type IV CRISPR/Cas system against plasmids carrying antimicrobial resistance genes introduces novel approaches to combat this issue [98]. The Type IV CRISPR/Cas system represents an advanced gene-editing technology that enables researchers to precisely locate and cure plasmids carrying antimicrobial-resistant genes, effectively preventing the production and spread of antimicrobial-resistant bacteria [99]. However, the research and application of this technology still encounter numerous challenges, including the intricacy of in vivo operation and potential safety risks. Thus, further research and exploration are imperative to ensure that its clinical application yields the anticipated outcomes [100]. Additionally, Redondo et al. uncovered evidence of cross-talk between type IV CRISPR/Cas systems and type I CRISPR/Cas systems, offering a straightforward explanation for the mystery of the absence of adaptive modules in type IV CRISPR/Cas systems [101]. Collectively, these findings redefine the role of CRISPR/Cas systems, prompt the expansion and reclassification of type IV CRISPR/Cas systems, and provide fresh insights into the biological function and evolution of these elusive systems.

In order to uncover the “mystery” of the IV-A CRISPR/Cas system, Cui’s team conducted research on the IV-A CRISPR/Cas system using the IV-A system of *Pseudomonas aeruginosa* to co-express effector proteins [102]. At this time, the effector protein and crRNA combine to form the effector complex CsfcrRNA. However, the characteristic protein Csf4 (CasDinG) of the system was not present in it. Biochemical experiments confirmed that CsfcrRNA could specifically recognize target dsDNA. The cryo-electron microscope structure of the CsfcrRNA and target dsDNA binding complex was analyzed by the single particle cryo-electron microscope method, revealing the molecular mechanism of the IV-A CRISPR/Csf system assembly, crRNA maturation, and target dsDNA recognition [102]. The Type IV CRISPR/Csf system has considerable potential for application as it is easier to deliver into cells and can identify a wider range of PAM sites, with a relatively large editing range and a wider application range [103]. In addition, the type IV CRISPR/Csf system can also be present in drug-resistant strains, so it can be correlated with drug-resistant genes. This would create an endogenous system that could potentially make it useful in combating the increasing spread of drug-resistant bacteria [104]. At the same time, as a multi-subunit complex, the type IV CRISPR/Csf system can couple other effector proteins, such as epigenetic modification enzymes and fluorescent proteins, at multiple locations, thus providing a wider range of applications [105]. Although this study revealed the mechanism by which the system targets DNA and recruits signature proteins, it is still unclear why this system exists primarily in plasmids, how it relates to antimicrobial genes in multidrug-resistant bacteria, and why the system prefers plasmids over chromosomal DNA as targets [102]. Nevertheless, this knowledge of these molecular details advances our understanding of the mechanism of IV-A CRISPR/Csf function and enables Csf complexes to be used in biotechnology.

All in all, the Type IV CRISPR/Cas system holds significant potential for the development of sequence-specific antimicrobials to address the growing problem of antimicrobial resistance. Unlike traditional antimicrobials, antimicrobials based on this system can precisely target specific plasmids carrying antimicrobial resistance genes, effectively eliminating them and reducing the spread of antimicrobial resistance genes. As research advances, it may become feasible for other plasmids within antimicrobial-resistant bacteria to eliminate antimicrobial-resistant plasmids carried by the resistant bacteria using appropriate methods through the IV-type CRISPR/Cas system, without the need for additional technologies. While the development and clinical application of these new antimicrobials will require extensive experimentation and validation, ongoing advancements in science and technology suggest that the Type IV CRISPR/Cas system could become a key tool in future antimicrobial development.

## 3. Delivery Modalities of CRISPR/Cas-Based Bactericidal Applications

**Figure 7:**
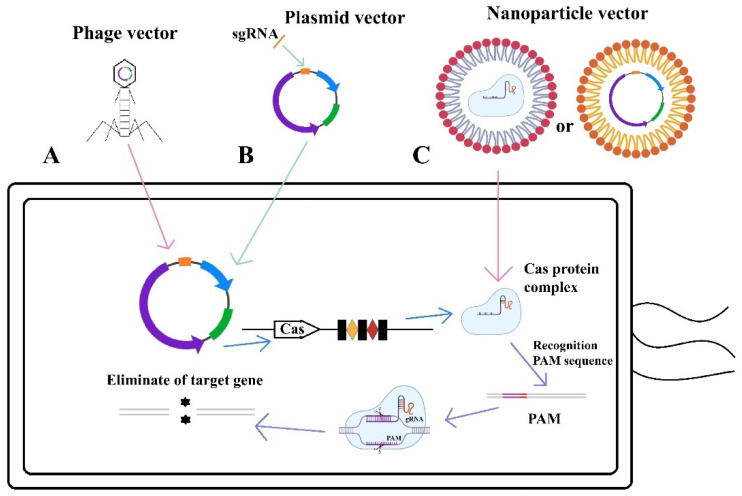
Delivery strategies for CRISPR/Cas-based antimicrobial agents. (**A**) Phage vector-based delivery (after delivery into the target cell, it is assembled into a complex to play a role); (**B**) Plasmid vector-based delivery (delivery into the target cell, then assembly into a complex to play a role); (**C**) Nanoparticle-based delivery (before being delivered to the target cell, it is assembled into a complex that plays a specific role, and it is also capable of effectively delivering plasmids).

The effective delivery of CRISPR/Cas-based antimicrobials to target bacteria is a fundamental challenge in utilizing this technology for combating and preventing antimicrobial resistance. The delivery methods for the CRISPR/Cas system can be classified into three main categories: physical, chemical, and biological approaches [106]. This section provides a concise overview of the various strategies employed to introduce the CRISPR/Cas system into target bacteria, with a particular emphasis on the use of phages, plasmids, and nanoparticles as delivery vectors (Figure 7).

### 3.1. Phage Vector

Phages naturally target bacteria and excel at injecting their DNA into host cells, making them a highly promising tool for delivering the CRISPR/Cas system [107]. Compared to other vectors, phage vectors possess a superior ability to invade host bacteria and accommodate larger DNA fragments [108]. Additionally, the nucleic acids encapsulated within phage proteins are more stable and less prone to degradation [109]. Phage vectors are being utilized to prevent and control the transfer of antimicrobial resistance genes using the CRISPR/Cas system. They can be integrated into the CRISPR/Cas system by encoding multiple proteins [108].

For instance, Kim et al. employed phages as vectors to introduce sgRNA and Cas9, specifically designed based on the conserved sequences of β-lactamase mutants, into a target strain. This approach successfully inactivated over 200 mutant pathogenic bacteria and restored sensitivity to β-lactam antimicrobials [110]. Similarly, Yosef et al. packaged a type I CRISPR/Cas3 system, including six Cas genes and various resistance genes related to ndm-1 and ctx-M-15, within lysogenic phages. This innovative method selectively dismantled antimicrobial-resistant plasmids, allowing host bacteria to regain sensitivity to multiple antimicrobials [111]. While phage-coated CRISPR/Cas9 can degrade antimicrobial resistance genes (ARGs) on plasmids without causing cell death, phage-coated CRISPR/Cas13a demonstrated significant bactericidal activity upon recognizing target ARGs [68].

However, a notable limitation of phage vectors is their narrower host spectrum. Researchers are exploring phage-based CRISPR/Cas antimicrobials to eliminate antimicrobial resistance plasmids or to kill resistant pathogens, though this delivery method still faces challenges in antimicrobial resistance therapy. Additionally, Hua et al. noted that the size of a phage’s capsid correlates with the size of its genome [112]. As a result, incorporating large CRISPR/Cas elements into the phage genome might impair phage assembly and replication. To preserve phage viability, it is essential to delete nonessential DNA fragments or to replace them with CRISPR/Cas elements. However, since many phages have not been thoroughly characterized, further investigation is necessary to understand the functions of phage genes before nonessential DNA can be removed [113]. Prolonged culturing of phages can lead to mutations that might alter their ability to target specific bacteria and diminish their therapeutic effectiveness [114]. In environments where multiple bacteriophages coexist, it becomes difficult to preserve the specificity and potency of each, as well as to prevent cross-infection [115]. Additionally, extended storage can result in a decrease in phage activity, highlighting the need for improved storage and preservation techniques [116]. During treatment, phages may interact with other medications or therapies, potentially impacting their stability and efficacy [117]. Furthermore, for certain drug-resistant bacteria, the effectiveness of a particular phage cannot be guaranteed, necessitating personalized treatment approaches that add complexity and uncertainty to the process [118].

Despite these challenges, it is believed that as our understanding of phage delivery mechanisms grows and existing methods improve, the host range of phage vectors will expanded and their delivery capabilities will be significantly enhanced.

### 3.2. Plasmid Vector

Plasmids can be classified into conjugative and non-conjugative types based on their horizontal transfer characteristics. Under natural conditions, conjugative-type plasmids transfer to recipient bacteria from donor bacteria via a self-encoded Type IV secretion system. In contrast, non-conjugative type plasmids do not require mediation from donor bacteria and can enter host bacteria through natural transformation [119]. Researchers have made strides in combating antimicrobial resistance by integrating sequences from the CRISPR/Cas system or components of this system into plasmid vectors, enabling targeted cleavage of antimicrobial resistance genes [120]. Conjugation serves as a vital pathway for bacterial gene transfer. Unlike phagocytic delivery, which necessitates specific receptors for recognition, plasmid uptake through conjugation does not require any such receptors [121]. Recipient bacteria harboring conjugation plasmids can further disseminate the CRISPR/Cas system to other bacteria, significantly broadening the potential applications of this technology in reducing antimicrobial resistance genes [122].

Rodrigues et al. demonstrated that plasmids engineered with the CRISPR/Cas system could reduce the occurrence of antimicrobial resistance in *enterococcal* populations in a sequence-specific manner [42]. Additionally, Kang et al. introduced plasmid vectors containing the pcascure system into carbapenem-resistant *Enterobacteriaceae*, effectively eliminating resistance genes such as blaNDM and blaKPC, thereby resensitizing these bacteria to carbapenems [123]. However, the delivery rate poses a significant limitation on the clinical application of the CRISPR/Cas system [124]. Other challenges in the plasmid conjugation process include low delivery efficiency and a narrow host range [125]. Many plasmid vectors rely on antibiotic selection pressure to identify transformed cells, but this method can lead to the dissemination of non-essential antibiotic resistance genes, presenting potential safety risks [126]. Additionally, within host cells, plasmid DNA may be degraded by incompatible enzymes or replicated unevenly, resulting in instability and the loss of recombinant plasmids [127]. By enhancing plasmid conjugation efficiency and Structural stability, we can expand the potential of plasmid vector-based CRISPR/Cas antimicrobials in preventing and treating antimicrobial resistance.

### 3.3. Nanoparticle Vector

Nanoparticle vectors generally refer to nanoscale carriers made from high molecular weight polymers or inorganic materials, characterized by their small size and exceptional ability to penetrate biological membranes [128]. With the rapid advancements in nanotechnology, a diverse array of nanoparticles—such as inorganic nanoparticles and cationic polymer-based nanoparticles—has been employed to efficiently transfer the essential components of the CRISPR/Cas system into target bacterial cells [129]. Nanoparticles can directly deliver Cas effectors and crRNA molecules into targeted bacterial cells.

When DNA is adsorbed or encapsulated by inorganic nanoparticles, it can overcome extracellular barriers and enter the cell through endocytosis, followed by the release of its cargo. This approach offers a novel delivery method for the CRISPR/Cas system [130]. Encapsulating the CRISPR/Cas system in nanomaterials enhances its stability, allowing it to be efficiently transported to its destination while maintaining high solubility for effective release and activity [131]. Currently, reports on nanoparticle delivery systems specifically targeting bacterial antimicrobial resistance genes within the CRISPR/Cas framework are limited. Various nanoparticles, including polymer nanoparticles, nanocrystals, cationic lipid nanoparticles, nanocages, exosome-liposome mixed nanoparticles, and zwitterionic amino lipid (ZALs) nanoparticles, are being explored as potential antimicrobial carriers [132].

For instance, Kang et al. demonstrated that a cationic polymer nano-CRISPR/Cas complex carrying the Cas9 protein and crRNA could be successfully introduced into the genome of methicillin-resistant *Staphylococcus aureus* (MRSA), effectively targeting and restoring sensitivity to methicillin [123].

Numerous studies underscore the potential of nanotechnology-based CRISPR/Cas delivery systems in combatting antimicrobial resistance [133]. However, the application of nanoparticles for delivering the CRISPR/Cas system is still in its infancy, with several unresolved challenges, such as achieving efficient targeted delivery to specific pathogens and optimizing assembly efficiency [134]. Although significant strides have been made in using nanoparticles to deliver the CRISPR/Cas system into target bacteria to address antimicrobial resistance, further research is needed to enhance efficacy and safety [135]. The distribution of nanoparticles within the body is influenced by various factors, including blood circulation, tissue penetration, and the immune response. These elements can lead to uneven accumulation of nanoparticles at different sites, ultimately impacting their therapeutic effectiveness [136]. Additionally, in vivo, its stability can be compromised by factors such as pH, temperature, enzyme activity, and salt concentration. Nanoparticles often accumulate in biological fluids, which may reduce their efficacy [137]. Furthermore, some nanoparticles are susceptible to oxidation or biodegradation in the body, impairing their ability to release payloads effectively [138]. The immune system may recognize nanoparticles as foreign substances, triggering an immune response that not only shortens their circulation time but can also lead to side effects, limiting their long-term application [139]. Overall, while nanoparticle carriers hold significant promise for drug delivery, addressing these limitations is crucial for advancing their clinical applications.

## 4. Innovative Antimicrobial Agents Derived from Diverse Fields

### 4.1. Antimicrobial Polysaccharides

In recent years, research into polysaccharides with antimicrobial properties has garnered significant attention due to their various pharmacological activities, including antioxidant, anti-tumor, and anti-diabetic effects [140]. These molecules can be found in a wide range of organisms, such as plants, animals, fungi, and bacteria, and they play crucial biological roles. Polysaccharides with antimicrobial activity, derived from different sources, are being explored as potential alternative therapies against multi-drug-resistant bacteria [141]. For instance, a study conducted in 2017 demonstrated that polysaccharides extracted from purslane exhibited inhibitory effects against acetic acid bacteria, *Escherichia coli*, and fungi. Notably, one high molecular weight polysaccharide showed greater antimicrobial activity than the others [142]. This finding aligns with previous reports suggesting that the antimicrobial efficacy of polysaccharides may be influenced by the specific monosaccharides that compose the polymer, as well as their molecular weight and structural characteristics [143].

Despite their broad spectrum of antimicrobial activity against various species, polysaccharides have not yet been developed into new drugs for treating multidrug-resistant infections. One contributing factor may be that much of the research on antimicrobial polysaccharides has been limited to in vitro studies, with a lack of clinical evidence supporting their safety and efficacy. Nonetheless, polysaccharides exhibit a potent inhibitory effect on pathogenic bacteria, positioning them as promising candidates for the development of novel antimicrobials.

### 4.2. Nano-Antimicrobials

Nanomaterials hold significant promise as antimicrobials, with various nanocrystals and mesoporous nanostructures being utilized as nanocarriers for antimicrobial agents [144]. Notable examples include photosensitizer nanoparticles (NPs), metal-organic frameworks (MOFs), covalent-organic frameworks (COFs), and semiconductor nanocrystals like nitrides, chalcogenides, and metal oxides, including TiO_2_, ZnO, BiVO_4_, and perovskites. These materials have demonstrated the ability to photocatalytically inactivate microorganisms under photoexcitation [145]. The electron–hole pairs generated in these systems can trigger chemical reactions with water or oxygen, leading to the production of reactive oxygen species (ROS) such as peroxides (O_2_^2−^), singlet oxygen (^1^O_2_), superoxides (O_2_^−^), and hydroxyl radicals (·OH). These ROS can induce oxidative stress and cause irreversible damage to microbial pathogens [146]. Overall, nanoantimicrobials have proven effective against a wide range of pathogens.

However, several challenges and limitations persist. Some nanomaterials may exhibit toxicity to normal cells, raising safety concerns for their clinical applications. Additionally, the stability and long-term effects of nanoantimicrobials within living organisms necessitate further investigation to ensure they do not degrade or accumulate in the body, potentially leading to health risks. While nanoantimicrobials are generally believed to be less likely to contribute to bacterial resistance, the emergence of resistant strains with prolonged use remains a concern.

### 4.3. Ribosomal Protein Antimicrobials

Some ribosomal proteins have demonstrated antimicrobial activity, presenting potential new sources of antimicrobial peptides (AMPs). Recent research has increasingly focused on multifunctional ribosomal proteins, which are capable of performing two or more unrelated roles within a cell or organism [147]. The scientific community is particularly interested in these AMPs due to their broad spectrum of antimicrobial responses against various infectious agents, including bacteria, viruses, parasites, fungi, and tumor cells [148]. For instance, Qu et al. showed that the ribosomal protein S15 interacts with bacterial membranes via lipopolysaccharides (LPSs) and lipoteichoic acid (LTA), leading to membrane depolarization and the induction of intracellular reactive oxygen species (ROS) in bacteria. This process can ultimately eliminate potential pathogens by triggering apoptosis [149]. Ribosomal proteins can generate ROS in bacterial cells, which can inflict damage on DNA, RNA, lipids, and proteins [150]. In conclusion, ribosomal proteins with antimicrobial properties are emerging as novel AMPs from both natural and synthetic sources.

Despite ongoing clinical trials exploring their use as AMPs, and research addressing their antimicrobial applications and selective toxicity towards cancer cells, these proteins have yet to find widespread industrial applications [151]. The mechanisms underlying the antimicrobial activity of ribosomal proteins remain not fully understood. Therefore, further investigations are essential to elucidate the mechanisms of action as AMPs, particularly regarding their specificity at the genus and species levels, and to evaluate their potential as effective antimicrobial agents.

### 4.4. Antimicrobials Targeting Bacterial Bioenergetics

Traditional small molecule inhibitors face significant challenges related to resistance in the treatment of infections and diseases, necessitating the exploration of new therapeutic targets. Most antimicrobials currently in use target only five fundamental cellular mechanisms and the rise of resistance has led to damage in these critical areas [152]. Bedaquiline (BDQ), a novel antimicrobial agent that targets the F1Fo-ATP synthase (an enzyme complex found on the cell membranes of specific bacteria that is essential for the cell’s energy metabolism, particularly during aerobic respiration), represents a promising new strategy for treating multidrug-resistant tuberculosis (MDR-TB) [153].

Bacterial bioenergetics play a crucial role in microbial persistence and are emerging as potential drug targets. However, while focusing on bacterial energetics offers the promise of developing new antimicrobials, it also presents numerous unforeseen challenges. The lessons learned from traditional antimicrobial targets have not yet translated into effective solutions. This new approach has significant potential to shorten treatment durations, address resistant pathogens, and eradicate persistent bacterial cells, but there is still much to understand [154]. The true diversity of pathogenic respiratory chains, at genetic, biochemical, and physiological levels, remains insufficiently explored, which limits our ability to target conserved mitochondrial complexes effectively. Additionally, the mechanisms by which organisms compensate for respiratory depression at both the substrate and oxidative levels are still not fully understood.

With advancements in genomics, metabolomics, and synthetic biology, there is hope to design drugs that specifically target bacterial metabolic pathways. This concept of precision medicine could minimize the impact on the normal microbiome and help mitigate the development of drug resistance.

## 5. Conclusions and Future Perspectives

Recent advancements in novel antimicrobials, particularly those utilizing sequence-specific approaches based on CRISPR/Cas systems, are promising. However, additional research is required to ensure their clinical viability and effectiveness [155]. There are still many challenges to overcome before CRISPR/Cas system-based antimicrobials can be safely and efficiently utilized to target antimicrobial resistance genes in natural microbial communities [156]. Sequence-specific antimicrobials based on the CRISPR/Cas system still have certain limitations. The targeting range of the CRISPR/Cas system is restricted by specific bacterial genome sequences, which means it may not be effective for some highly variant or emerging drug-resistant strains. Just like other antimicrobials, bacteria may gradually develop resistance to CRISPR/Cas antimicrobials, potentially limiting their long-term clinical use.

Further research and evaluation are necessary to ensure the long-term safety and potential side effects of the CRISPR/Cas system in humans for clinical applications. What is more, there are numerous clinical application challenges that need to be addressed, such as dose control, treatment duration, and other issues. Therefore, although CRISPR/Cas system sequence-specific antimicrobials offer many potential advantages, they are not yet available for large-scale clinical use. Related future research and technological developments should focus on addressing these issues. This includes further research and development to broaden the targeting range to cover more bacterial strains and resistant variants, optimizing the structure and function of CRISPR/Cas system antimicrobials to improve their targeting accuracy and efficiency, enhancing their effectiveness, reducing adverse effects, and realizing their application in more realistic microbial communities. Designing more durable antimicrobials to minimize the development of bacterial resistance to CRISPR/Cas system antimicrobials is also crucial. Furthermore, conducting more clinical trials, monitoring to evaluate the safety and effectiveness of CRISPR/Cas antimicrobials in clinical applications, and providing data support for their further optimization will promote the application of CRISPR/Cas systems in the field of antimicrobials. CRISPR/Cas-based sequence-specific antimicrobials hold significant promise in combating antibiotic-resistant bacteria. However, their long-term use comes with potential risks. Even precise CRISPR/Cas systems can drive bacterial mutations as they pressure bacteria to evade bactericidal effects, and bacteria might develop resistance through mutations in target genes [157]. Additionally, while CRISPR/Cas systems are designed to be specific, there is a risk of unintentionally affecting non-target bacterial populations. This can disrupt the balance of microbial communities, potentially leading to the proliferation of resistant strains and altering the overall composition of bacteria in the environment, which may have health implications [158]. Currently, comprehensive research on the long-term effects of CRISPR/Cas systems is lacking, leaving questions about their impact on microbial ecology and human health unanswered [159]. Furthermore, bacteria may develop new strategies to escape CRISPR targeting, such as modifying the sequences of CRISPR target sites or producing enzymes that inhibit the CRISPR machinery, further accelerating the emergence of resistance [160]. Utilizing CRISPR/Cas systems naturally present on plasmids may help avoid some of the problems associated with releasing genetically engineered organisms, but understanding the consequences of releasing any DNA elements on a large scale will be crucial for the sustainable and risk-free implementation of the technology [125].

The author believes that CRISPR/Cas-based sequence-specific antimicrobials can be combined with other antimicrobial strategies, such as traditional antimicrobials, immunotherapy, etc. For instance, by combining CRISPR/Cas-based sequence-specific antimicrobials with traditional antimicrobials, it is possible to precisely target and eliminate specific resistance genes while simultaneously inhibiting bacterial growth. This approach helps mitigate the emergence of antibiotic resistance. Alternatively, the research advancement of CRISPR/Cas-based sequence-specific antimicrobials can be integrated with other technologies, such as the development of new delivery vectors and the improvement of existing ones to augment delivery efficiency. The emergence of nanotechnology may offer a novel approach for CRISPR/Cas system delivery and appropriate nanocarriers could significantly enhance delivery speed and delivery volume. In addition, it can also be combined with other systems, such as the λ-Red system. These measures could reduce the onset time of action or bolster antimicrobial effects while mitigating resistance development and minimizing CRISPR/Cas-based sequence-specific antimicrobial usage. Once research and application are mature, these methods can be employed in personalized treatment, leveraging the CRISPR/Cas system to design highly specific antimicrobials tailored to particular bacterial strains, tailored treatment based on the individual circumstances of patients, thereby improving therapeutic efficacy. The use for preventive treatment, for example, a specific antimicrobial could be designed to target a precise pathogen in a specific environment, thereby reducing the risk of infection. Furthermore, to be eco-friendly, CRISPR/Cas systems can be utilized to design antimicrobials that are human and environmentally friendly, thus lessening the environmental impact of antimicrobials. CRISPR/Cas-based sequence-specific antimicrobials can be easily reprogrammed to target specific genes, greatly enhancing the feasibility of implementing related technologies. This advancement could be instrumental in addressing the escalating issue of antimicrobial resistance and may aid in preserving or reinstating the antimicrobial activity of antimicrobials.

Novel antimicrobials, particularly those based on CRISPR/Cas technology, are poised to play a crucial role in the future of antimicrobial therapy. These innovative treatments not only target specific pathogens but also minimize the impact on surrounding microbes, thereby reducing the likelihood of resistance development. As our understanding of the microbiome deepens, researchers are increasingly recognizing the importance of maintaining its balance. The emergence of new antimicrobial agents, especially those leveraging gene-editing technologies, allows for precise intervention against harmful bacteria without disrupting beneficial flora. This targeted approach aids in restoring the body’s natural defense mechanisms and lowers the risk of secondary infections and other complications. Moreover, the rapid advancement of CRISPR/Cas technology enables us to adapt and refine the targeting capabilities of these antimicrobials in real-time, responding swiftly to the challenge of drug-resistant bacteria. This adaptability acts as a safeguard against future healthcare challenges. However, the successful translation of these innovations into clinical practice will require collaboration among multiple stakeholders to ensure their safety and efficacy, ultimately enhancing patient health outcomes.

## Data Availability

Data sharing is not applicable to this article as no datasets were generated or analyzed during the current study.
